# Development of a High Precision Telescopic Instrument Based on Simultaneous Laser Multilateration for Machine Tool Volumetric Verification

**DOI:** 10.3390/s20133798

**Published:** 2020-07-07

**Authors:** Juan José Aguilar, Raquel Acero, Francisco Javier Brosed, Jorge Santolaria

**Affiliations:** 1 Department of Design and Manufacturing Engineering, University of Zaragoza, María de Luna 3, 50018 Zaragoza, Spain; jaguilar@unizar.es (J.J.A.); fjbrosed@unizar.es (F.J.B.); jsmazo@unizar.es (J.S.); 2 Instituto de Investigación en Ingeniería de Aragón (I3A), 50018 Zaragoza, Spain

**Keywords:** machine tool, multilateration, telescopic system, interferometry, kinematic coupling

## Abstract

This paper presents the design of a high precision telescopic system consisting in three lines, with measuring principle based on simultaneous laser multilateration. The system offers the high precision of the interferometer systems and allows the autonomous tracking of a sphere joined to the spindle nose of the machine tool by simultaneous contact of all the lines. The main advantage of the system is that it allows data capture to be carried out in a single cycle thanks to simultaneous operation with at least three telescopic arms using a novel multipoint kinematic coupling. This results in a significant reduction of the time taken for data capture and improves measurement accuracy due to avoiding the effect of temperature variations between cycles and machine tool repeatability. The work explains the working principle of the system, its main components, and the design parameters considered for the development of the system. The system is simple to operate, compact, agile, and suitable for the verification of small- or medium-sized machine tools with linear and/or rotary axes.

## 1. Introduction

Modern manufacturing technology demands efficiency and accuracy in machining, handling, and measuring processes. For this purpose, traceable dimensional metrology techniques assure the consistency and repeatability of manufacturing and measuring equipment. Machine tools (MTs) calibration and verification processes are a clear example of the research field where important achievements have been obtained. The gradual implementation of open architecture control software installed in machine tools compensates the MT’s geometrical errors that could be obtained in the volumetric verification, thereby improving the accuracy of the machine. Traditionally, machine tools and coordinate measurement machines (CMMs) accuracy have been ensured verifying each machine’s axes in a process known as geometric verification. Each geometric error could be measured individually for a particular position in the MT workspace [1] with direct measuring techniques according to ISO 230-1:2012 [2]. They can be classified into three subgroups, specific methods with standard systems, specific methods using the linear propagation of the laser and its wavelength as a reference, as well as multidimensional artifacts such as telescopic bars [1,3,4] or specific methods based on gravity such as levels [5]. The errors characterized by this verification technique are obtained from the positioning of a system of known dimensions in different positions of the MT, but the influence of the error identified in that position cannot be extrapolated directly to the rest of the machine working volume.

Nevertheless, due to challenging industry requirements, which demand parts with high accuracy in different sizes and increasing productivity, volumetric verification has been extended progressively as an efficient and reliable verification technique. With indirect measuring methods, the combined influence of MT geometric errors is calculated considering the MT kinematic model and the axis movement [6,7,8]. Therefore, volumetric verification determines the behavior of the machine in its entire workspace in less time than geometric verification, providing an overall correction of the combined effect of all geometric errors of the MT, thereby minimizing its volumetric error. When the geometric errors are characterized, their influences are compensated using MT software control. The correction provides global compensation of the geometric errors which finally improves the accuracy of the machine. Consequently, the approximation function of each geometric error is not required to coincide with its physical meaning. The accuracy of the geometric error approximation functions obtained in the volumetric verification depends on the measurement system characteristics, the techniques, and the number of equipment selected. Different measuring equipment are used for MTs volumetric verification, such as ball bars [9,10,11,12], laser tracers [13,14,15], and laser trackers [16,17,18].

One of the most extended ones is the laser tracker (LT), which is a portable measurement system that provides the position of a point in spherical coordinates [19]. This position is determined by a laser interferometer to measure relative distances (d) and optical encoders for measuring azimuth angle (*θ*) and elevation (φ) of the laser beam. A laser tracker, like any measurement system, has a measurement uncertainty because of its design, structural configuration, and environmental conditions in which the measurement is made [20,21,22,23,24]. In addition to these sources of error, there is the influence of the uncertainty of additional measurement elements such as retro-reflectors and the use of active targets in data capture, in case they are incorporated into the verification [25].

The different sources of error of laser-tracking based measurement systems used in verification of manufacturing systems are divided into two main groups of errors: systematic errors, which can be compensated, and random errors. Both systematic and random errors cause the measurement to be affected by measurement uncertainty. Gallagher divided the different sources of error into angle encoders, tracking system, perpendicularity, measurement distance, and beam alignment [26]. Teoh classified them into errors coming from environmental factors, errors in measurement acquisition, and errors as a consequence of the approximation and simplifications made [27]. All these error sources will influence the laser tracker interferometer, the motors with angle encoders, and the position sensing detector (PSD), thus affecting the measurement accuracy.

Currently, the improvement of accuracy at the points of capture because of the design, manufacture, and components of the LT is addressed from two different approaches. The first approach is based on the study of the different errors that affect the accuracy of each LT through an analysis of its structural configuration and its inherent errors, systematic errors. The influence of these errors is attempted to be compensated through the LT control software. The second approach is based on the improvement of the captured data by applying measurement noise reduction techniques that affect the capture data, random errors, by measuring them from three or more different positions [28,29,30,31,32]. The two approaches are fully complementary.

The influence of random errors could be reduced by applying the multilateration technique that uses the radial information provided by the measurement of a point from three or four different positions to determine the coordinates of the measured point without information from the angular component of the LTs. The measurement error reduction obtained by multilateration is determined by the number of LTs used, the spatial distribution of the LTs relative to the MT’s workspace, the relative position among the LTs, and the measurement strategy [33,34]. Other solutions for small and medium-sized MT verification including consecutive measurement cycles by multilateration have been identified in [35,36]. They propose both single telescopic systems, which track a retroreflector attached to the MT’s spindle nose by means of a kinematic coupling.

This paper presents the design of a high precision telescopic system consisting in three or more lines, with measuring principle based in simultaneous laser multilateration. The system offers the high precision of the interferometer systems and allows the autonomous tracking of a sphere joined to the spindle nose of the MT by simultaneous contact of all the lines using a novel multipoint kinematic coupling. It allows data capture in a single cycle because of the simultaneous operation of the telescopic arms. The system developed provides flexibility and measuring process time similar to those of the LT but improves its precision until levels close to those of the laser interferometry, avoiding the effect of temperature variations between cycles. The system will be oriented to the verification of small and medium-sized machine tools but it could also be applied to the verification of coordinate measuring machines (CMMs), articulated arm coordinate measurement machines (AACMMs), and robots. Finally, the design is validated with the assembling of a prototype.

## 2. Design of the High Precision Telescopic System

### 2.1. Working Principle

In this paper, we present the design of a multi-telescopic arm system for volumetric verification of small and medium-sized MTs based on high precision simultaneous multilateration. The integration of three or more telescopic arms simultaneously allows the volumetric verification of manufacturing and measuring systems in a fast and economical way. It aims the use of these machines in processes that require higher performance such as high precision manufacturing, precise measuring and handling operations, or precise and traceable process inspection tasks at the machine. The system is simple to operate, compact, easy to assemble, agile, and suitable for the verification of small or medium-sized machine tools with linear and/or rotary axes.

The main advantage of the system is that it allows data capture in a single cycle thanks to the simultaneous operation with at least three telescopic arms that are registered simultaneously at a single point. The system enables measuring the position of a sphere fixed in the machine spindle nose from at least three different positions of the MT table by means of multilateration in a single cycle. From each position, the displacement to the head is measured with a telescopic system that includes an interferometer equipment inside. Therefore, there is a significant reduction of time in the data capture process as only one cycle is required as opposed to the three, four, or more required with other available systems. This is due to the strong limitation in the positions from which the measurement systems can capture the data. Additionally, the accuracy of the results is improved by simultaneously capturing the necessary information in a single multilateration cycle, eliminating the influence of thermal variations between cycles and machine tool repeatability. The measurement of each point in the working volume is performed statically to increase precision avoiding the lack of synchronization between the measurements of the three lasers.

### 2.2. Main Components of the Telescopic System

The system integrates several components that are designed so that the multiple telescopic arms could follow the movement of the MT spindle, measuring the same point simultaneously, and without interfering with each other. In particular, the system has been equipped with a new multipoint kinematic coupling system on each telescopic arm that enables it to follow the movement of the spindle nose without colliding with other elements or saving collisions, and with enough force to drag the telescopic arms in their movement.

**Figure 1:**
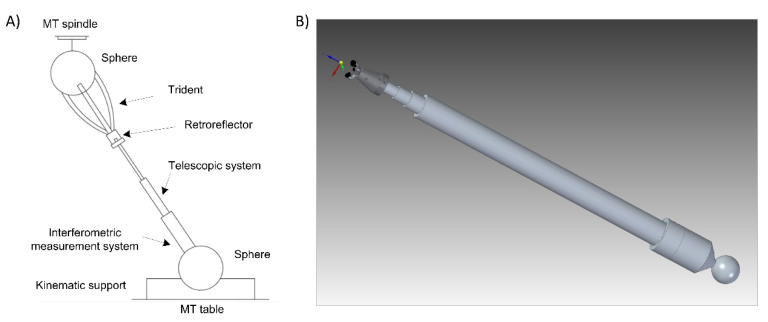
Extensible telescopic system (**A**) initial concept, (**B**) first prototype development.

The main components of the verification system are:

- Telescopic arms. The system designed comprises initially three telescopic arms. Each telescopic arm (Figure 1) has a conventional kinematic support at one end that allows it to register and rotate with respect to a point on the MT table. A coupling based on a ferromagnetic sphere has been defined, which is attached to one end of the telescopic arm with a conventional magnetic holder fixed to the table. In this way, the magnetic holders allow the free rotation of the sphere through a ball-joint type with the least possible friction and preventing all the displacements.
  For the connection of the telescopic arms to the MT, a common ferromagnetic sphere is used, which is attached to the MT spindle nose. Each arm rotates freely with respect to its kinematic coupling with the minimum possible friction to enable the required movements without collision among the fingers of the multipoint kinematic couplings (tridents). In addition, the arms can be extended with their telescopic system with the minimum possible friction and have sufficient rigidity to enable high precision measurements.
- Trident: on the other side of each telescopic arm, a multipoint kinematic coupling allows the registration and rotation with respect to another point. In this case, it enables several telescopic arms to be registered and rotate simultaneously and independently with respect to the same point, see (Figure 1). Thanks to the design of this kinematic coupling, each arm is in contact with the ball anchored to the machine spindle nose. The coupling is a trident system consisting of three fingers that contact the sphere at three points, with the arms forming a desirable relative angle of 90° from the center of the sphere. This allows continuous and precise registration with respect to the center of the sphere and the coexistence with the other tridents of the arms avoiding possible collisions. The connection between the trident and the sphere anchored to the MT spindle nose is made by means of magnetic systems. They are located on the upper part of each trident’s finger, enabling the required free movement. The magnetic systems make it possible not only to maintain an attraction force with respect to the fixed sphere to avoid its separation, but also generate a repulsion force among the fingers of different tridents to avoid their contact or collision. In this way, their relative rotation is permitted, adjusting themselves to the required trajectories without hindering its operation.
- Laser measuring system. The telescopic arms have a laser measurement system by interferometry with the emitting head at one end and a retroreflector at the end close to the sphere fixed to the MT spindle nose (Figure 1). The laser system, with characteristics summarized in Table 1**,** measures directly the relative displacement between the sphere attached to the MT spindle nose and the sphere fixed in the magnetic holder located on the MT table. It measures in an incremental way from a reference position that is materialized in a calibration gauge [37] by means of a fixed magnetic sphere-holder that locks the position of the sphere fixed to the instrument. The gauge is designed with several kinematic supports to obtain a repeatable positioning of a movable sphere that materializes the different calibration lengths of the telescopic arm.

**Figure 2:**
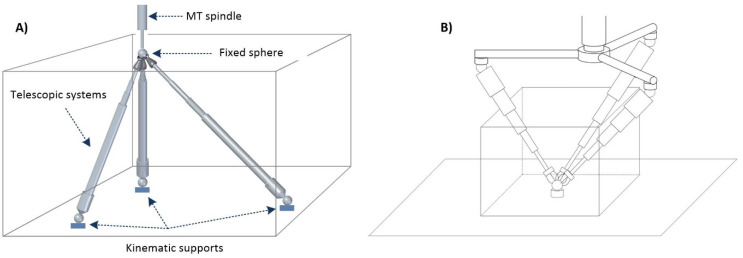
Telescopic system fixation concept, (**A**) Positioning on machine tool table, (**B**) Positioning on machine tool spindle nose.

The system can be positioned on the machine tool in two different configurations, either the traditional position on the machine table (Figure 2a) or in the reverse position on the machine tool spindle nose by means of a specific clamping system (Figure 2b). Figure 2a shows the three kinematic supports on the MT table and the three telescopic arms, each of them placed in such a way that they can swivel with respect to its respective kinematic support. In this configuration, the measuring system is located in the part closest to the table and the trident is in contact with a sphere attached to the MT spindle nose. In the case of the arrangement according to Figure 2b, the three kinematic supports are connected to the MT spindle nose with a clamping system consisting of three auxiliary arms. In this way, the telescopic system is suspended from the MT spindle by the clamping system. This arrangement improves the measurement process of rotary axes associated with the table movement. The configuration presented in this work is the traditional one shown in Figure 2a, with the positioning of the kinematic supports on the MT table and the fixed sphere attached to the MT spindle nose.

### 2.3. Design Parameters

For the definition of the main design parameters of the complete verification system, two different machine tool working volumes were considered, a medium-sized MT (machine tool 1) and a small-sized MT (machine tool 2) with the specifications included in Table 1. The technical characteristics of the interferometry equipment integrated in the system are listed in the same table.

#### 2.3.1. Telescopic Systems Angle

**Figure 3:**
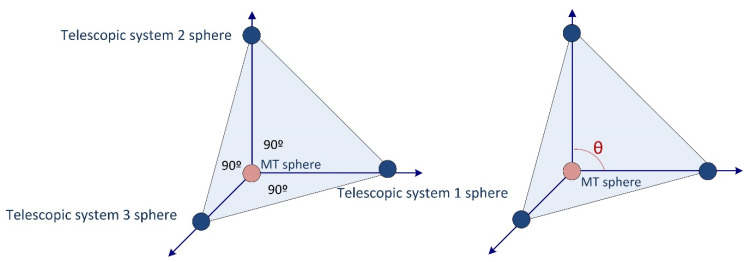
Triangular tetrahedron configuration for the telescopic systems.

Imaginarily, the system with the three telescopic arms creates a tetrahedron whose vertices are formed by the three centers of the ferromagnetic spheres at the ends of the telescopic arms located on the magnetic holders at the MT table, and the center of the sphere connected to the MT spindle nose where the three arms’ tridents are registered (Figure 3).

The measuring system improves the accuracy of the multilateration when it acquires the geometry of a tri-rectangular tetrahedron. An arrangement close to this geometry at the center point of the working volume will be convenient to locate the verification area. The tri-rectangular tetrahedron contains four faces, and three of these faces are made of right triangles. The right angles of these triangles coincide at the same vertex where the fixed sphere is located, whether attached to the MT spindle nose (Figure 2A) or placed in the kinematic support on the MT table (Figure 2B). The remaining triangle is an equilateral triangle with the three spheres of the telescopic systems placed in the corresponding kinematic mountings. Since the centers of the spheres are located at the vertices of the triangle, they are equidistant from the center of the triangle and the angle between each pair of telescopic systems is 90°. When the working point travels through the MT working volume, the angles (*θ*) between the telescopic systems vary.

One of the main parameters for the design of the trident are the limit values of the angle (*θ*) between each pair of telescopic systems. It is therefore necessary to study the maximum and minimum values of these angle to ensure the correct verification of the positions in the working volume defined for the two types of machine tools under study.

**Figure 4:**
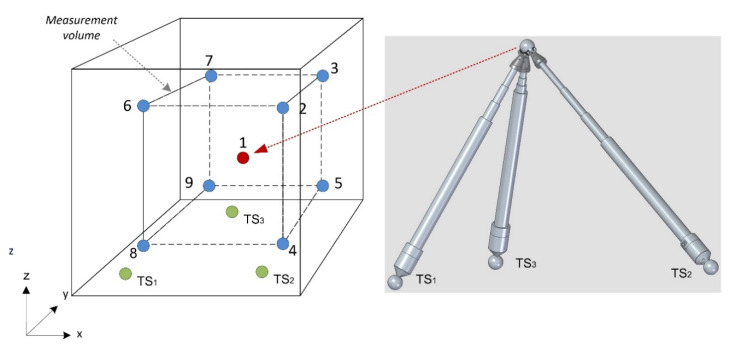
Telescopic system verification points sequence in the MT working volume.

In this case, a simulation of the angles between telescopic systems (maximum and minimum) has been carried out for machine tool 1 (see Table 1). Nine fixed verification points have been defined to be verified in the machine working volume, forming a parallelepiped. These points are verified simultaneously by the three telescopic systems TS_1_, TS_2_, TS_3_ with kinematic support and sphere pairs located on the machine tool table, see Figure 4. The verification sequence begins with point 1, where the machine tool head is positioned. The nine points defined in the machine’s measuring volume are checked in correlation order from 1 to 9 according to the scheme in Figure 4, dragging the fixed reference sphere the telescopic arms that are registered to the sphere by the respective tridents.

The results of the simulation carried out for the medium-sized machine tool 1 for the angles (*θ*_12_, *θ*_23_, *θ*_31_) formed between the three telescopic systems in the verification sequence of the nine points defined in the MT verification volume can be seen in Table 2. This angle is a critical value for designing the body of the trident and preventing its collisions with other tridents during the verification process of the MT.

**Figure 5:**
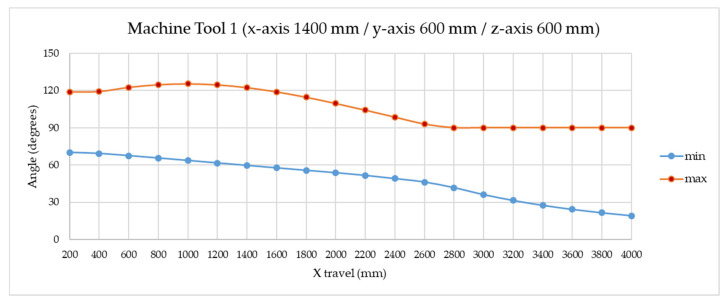
Angle between telescopic arms variation with x-axis travel in machine tool 1.

**Figure 6:**
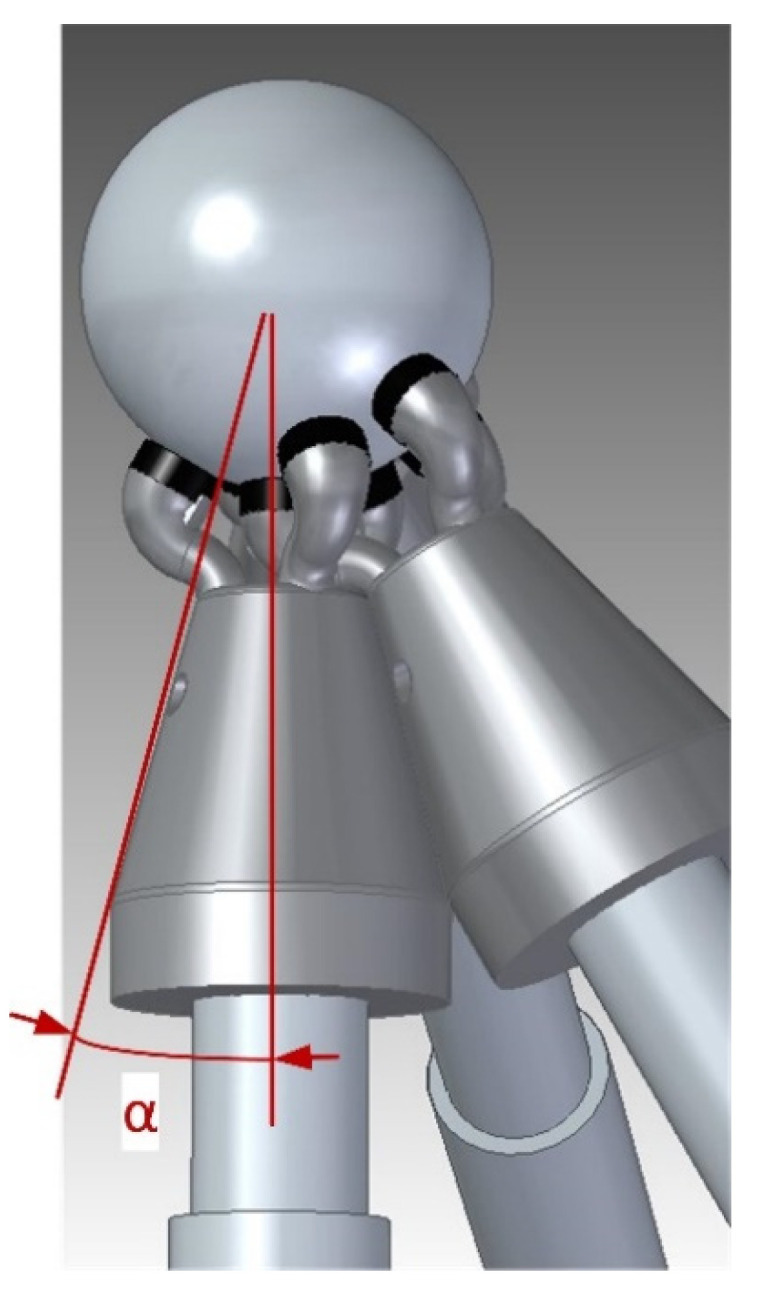
Trident’s body angle and fingers spatial disposition.

This simulation has been extended by varying the x-axis with the greatest travel between 200 and 4000 mm to analyze further verification scenarios. Figure 5 shows how in the normal x-travel range values, between 600 and 2000 mm, the angles are centered at 90° (desirable configuration), with maximum and minimum values around 120° and 60°, respectively. When the travel increases, the minimum angle decreases. If the body of the trident has an angle (*α*) of 15°, it is possible to work in a single multilateration cycle with machine (x/y/z) travels up to 3200 × 600 × 600 mm. This is the angle value selected in the trident’s design as shown in Figure 6. In addition, to avoid collisions among the fingers, the tridents have been designed starting from the front surface and permitting them to fit inside each other, adopting spatial dispositions that prevent collisions. A larger x-axis travel would result in unbalanced special machines requiring two overlapped measurement cycles.

#### 2.3.2. Telescopic System Arms Length

As stated in Section 2.2, the arms have a telescopic configuration that allows them to be extended with the minimum possible friction and sufficient rigidity to enable the required precision movements. When setting the main design parameters of the telescopic system arms, the MT verification volume indicated in Table 1 was considered. We designed a telescopic arm with precision cylindrical tubes made of anodized aluminum EN AW 6061/6060, tolerance h8.

**Figure 7:**
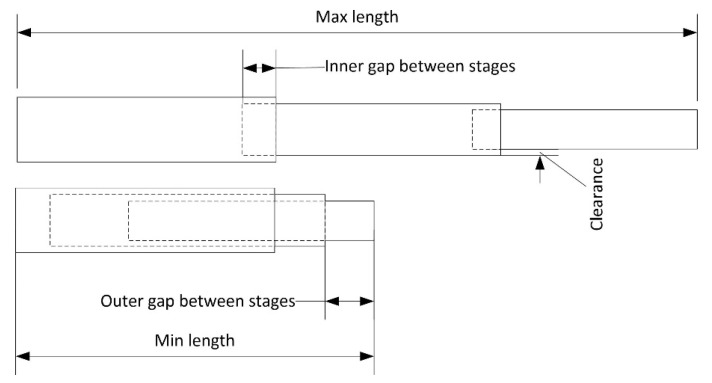
Design parameters in the telescopic system.

Some design concepts of the telescopic arm are introduced below in Figure 7, such as the maximum and minimum length of the system, as well as the internal and external gaps between the stages the telescopic system is divided into.

- Maximum (*L_max_*) and minimum length (*L_min_*): length that reaches the telescopic arm in its most extended and retracted position.
- Effective measuring range (Δ_L_): range that the telescopic arm can be extended. It is calculated as the difference between the maximum and minimum length.
- Stages (*n*): each of the sections that the telescopic arm is divided into, which can be extended by moving them with respect to the previous one. They are numbered from the stage that in the retracted position holds all the others.
- Stage length (*L_i_*): length of each of the n-stages of the tube. It is the same in all of them.
- Effective stage length (*L_ief_*): difference between the stage length (*L_i_*) and the inner gap between stages (*Inner_gap_*).
- Inner gap between stages (*Inner_gap_*): minimum length that a stage remains embedded in the previous stage to minimize the radial clearance between stages.
- Outer gap between stages (*Outer_gap_*): length that exceeds one stage in relation to the previous one when it is retracted.
- Clearance (*c*): gap between stages in the radial direction.

The more stages and the longer these stages are, the longer the telescopic system will be. For the same maximum length, very long stages will make the minimum length not so reduced, since the length of the first stage collecting the others will be greater and will limit the minimum length to be reached. On the other hand, it must be taken into account that a greater number of stages may result in a bigger clearance due to the cumulative gap of the stages. Likewise, a high number of stages means that the last one may require a too small profile to allow its retraction in the previous ones. This may make it more likely to suffer deformations that result in a bending deflection of the system.

In order for the stages to remain connected to each other as the telescopic arm expands, it is unfeasible for one stage to project completely from the previous one, as the element would be dismantled and lose its function. To maintain this contact between stages, low friction and low wear plain plastic bearings designed for the lowest coefficients of friction while running dry and low stick slip tendency are used. Depending on the connecting element between stages, there will be a space shared by the two consecutive sections of between 15% and 25% of their length, named as the inner gap between stages in Figure 7. This internal space exists even at the maximum extension of the telescopic element, so its maximum action range will be less than the result of multiplying the stage length by the number of stages. This also applies for the telescopic arm in its most recessed position, where there is also a small external space between stages, which means that the stage is not fully inserted into the previous one (Figure 7).

**Figure 8:**
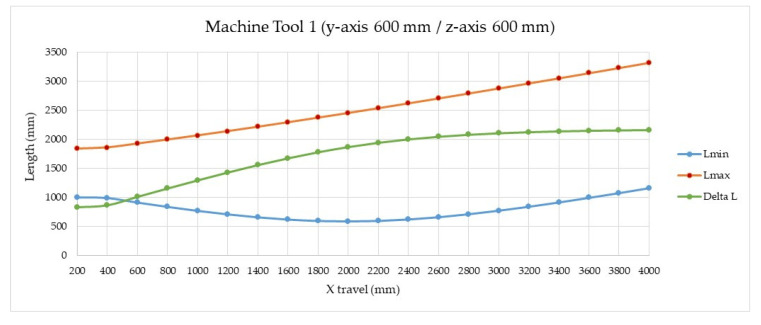
Telescopic system length simulation: max–min length and effective system length (Delta L).

We performed an analysis to define the main design parameters of the telescopic arm, maximum length (*L_max_*), minimum length (*L_min_*), effective measuring range (Δ*_L_*), and the number of stages necessary (n). The simulation was carried out for machine tool 1 (see Table 1) proposing for the verification of its working volume a telescopic arm with 5 stages (n) with 500 mm of stage length (*L_i_*) and 400 mm of effective stage length (*L_ief_*). The calculated maximum (*L_max_*) and minimum (*L_min_*) lengths of the telescopic arm are 2254 mm and 654 mm respectively, obtaining an effective measuring range (Δ_L_) of 1600 mm. Figure 8 presents the calculations made for the case of machine tool 1 (see Table 1) where the travel of the x-axis varies between 200 and 4000 mm. It could be concluded that to verify machines with a x-travel greater than 1400 mm, it would be necessary to add more stages to the telescopic arm.

For the verification volume in machine tool 2 (see see Table 1), it is intended a telescopic arm with 5 stages, with 223.5 m of stage length (*L_i_*) and 146.3 mm of effective stage length (*L_ief_*). The calculated maximum (*L_max_*) and minimum (*L_min_*) lengths of the telescopic arm would be 960 and 375 mm respectively, obtaining an effective measuring range (Δ_L_) of 585 mm.

An important fact analyzed in the work is the identification of the error sources that can produce a misalignment in the telescopic system, causing consequently a misalignment in the laser interferometry system that would prevent the correct measurement. This misalignment may be due to the existing gap between the stages of the telescopic system and to the deflection of the telescopic system itself in its extension. The relative movement of the telescopic arm stages, results in the existence of a clearance (c) between the elements due to tolerances, manufacturing defects, or merely due to the design of the telescopic system itself. Misalignment derived from the bending may be caused by several factors such as the tube manufacturing material properties, the tube thickness, or the weight supported at the end of the telescopic element. Experimental testing and finite element analysis of the system have been carried out to properly design the system and to evaluate the effects of the system bending and clearances. A target value of less of 1.5 mm for radial displacement of the trident at the maximum extension position of the telescopic arm has been settled. Considering this displacement and using a solid glass corner reflector of 6.35 mm diameter and 5 mm height, model Newport BGR-6.35, the laser system can measure correctly making negligible errors despite slightly lowering in the return signal.

#### 2.3.3. Tridents Magnets and Sphere Diameter Relation

As mentioned above, one of the fundamental elements of the telescopic system is the multi-point kinematic coupling that allows the three telescopic arms to be registered and rotated simultaneously and independently with respect to the center of the fixed sphere anchored to the MT spindle nose. The trident consists of three fingers that contact the sphere at various points, forming a given relative angle from the center of the sphere. The trident must always be able to register precisely on the sphere and avoid possible collisions between the three tridents. For this purpose, magnetic systems are placed on each of the fingers of the trident to allow the free movement of the arm trident. The most important design parameters of the trident are the finger magnets diameter and force, as well as the geometry of the trident’s fingers. Both have a direct relation to the diameter of the fixed sphere.

**Figure 9:**
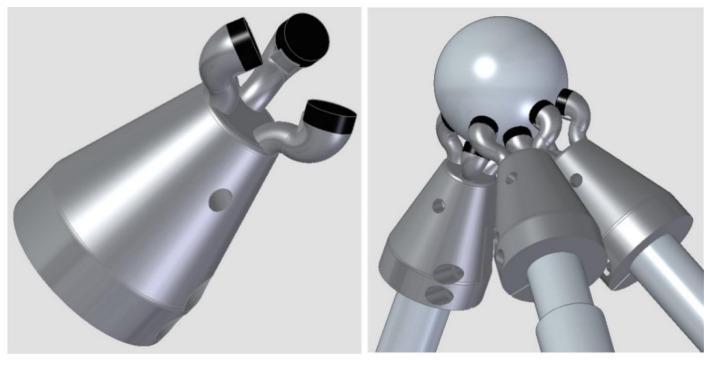
Trident design with magnetic fingers for single telescopic system.

The magnetic system coupled to each finger allows not only to maintain an attraction force with respect to the sphere avoiding the tridents separation from the sphere, but also generates a repulsion force among other tridents’ fingers. This will avoid their contact or collision and allows their relative rotations to fit the necessary verification trajectories without preventing their operation. A minimum space between fingers is required to ensure the correct movement of the system. A design of the trident with the magnets arranged at the ends of the fingers is shown in Figure 9. An example of the positioning of the three tridents on the fixed sphere is also presented in the same figure.

To correctly design this critical part of the telescopic system, the variation of the minimum percentage of collision between the tridents’ fingers by varying the diameter of the fixed sphere (D) and the trident’s finger diameter (d) was simulated with the verification of nine fixed points with the verification sequence described in Section 2.3.1. At each point, the percentage of positions without collision is calculated by rotating the tridents for the combinations of pairs of arms and the minimum is calculated. The analysis was performed for both the medium and small-sized machine tools 1 and 2, (see Table 1).

At each point, the percentage of collision-free positions is calculated by rotating the tridents two by two around the axis of the telescopic arm and the minimum percentage of collision-free positions is calculated. This calculation is performed first between telescopic arms 1 and 2, then between telescopic arms 2 and 3, and then between telescopic arms 3 and 1. For each pair of arms, for example arms 1 and 2, and starting from an initial position of the two arms, it is analyzed if the fingers of the tridents collide when the distance between the contact points of the fingers and the sphere is less than a reference value. This value is calculated out of the diameter of the fingers and the diameter of the ball (when the diameter of the ball is very large in relation to the diameter of the fingers, this value is only slightly less than the diameter of the fingers).

This calculation is repeated in two loops. First, the position of arm 1 is fixed and the 2 is rotated degree by degree, adding up how many of the 360 positions present a collision. Subsequently, arm 1 is rotated one degree and the procedure is repeated with arm 2. This continues until the trident of arm 1 rotates 360°. The percentage of positions with collision in the cumulative sum of positions with collision divided by the total number of positions (360 × 360). This procedure is repeated for arm combinations 2 and 3 and then 3 and 1. Three values of percentage of collisions are obtained and the minimum is calculated for that point of the working volume of the machine tool in study.

Three fixed sphere sizes were simulated, with 1, 1.5, and 2 inches diameter and the trident’s fingers diameters ranged from 3 to 11 mm.

In the case of the medium-sized machine tool 1, it is experimentally observed that a minimum non-collision percentage of 50% is advisable for the system to operate without the kinematic couplings being released from the fixed sphere attached to the MT spindle nose, in measurement processes with rapid feed x/y/z of about 20 m/min. When working at lower feeds, the trident turns occur more slowly, there are less inertial forces, so a lower minimum collision percentage can be accepted without releasing the tridents from the sphere. In the case of working at higher feed, the opposite happens accordingly and a higher minimum collision percentage is necessary.

**Figure 10:**
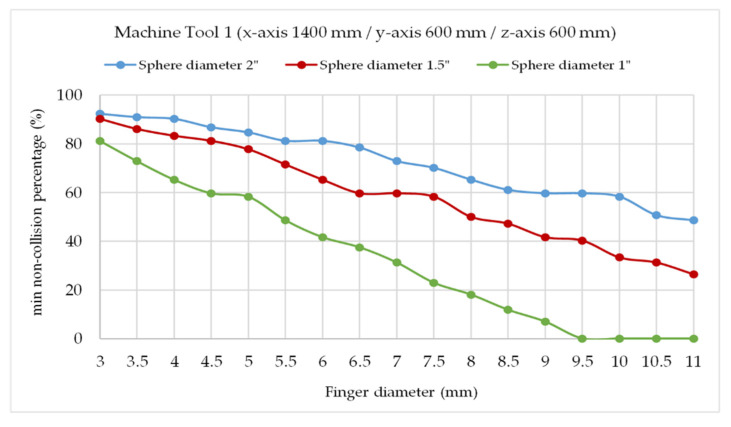
Finger diameter for min 50% non-collision positions in machine tool 1 (sphere diameters 1′′, 1.5′′, and 2′′).

Under these conditions that ensure 50% non-collisions at the verification positions, Figure 10 shows that the maximum finger diameters calculated as a function of the fixed sphere size are 10.5, 8, and 5 mm for reference sphere diameter values of 2′′ (50.8 mm), 1.5′′ (38.1 mm), and 1′′ (25.4 mm), respectively.

In the first prototype design, we considered a fixed sphere of 1.5′′ diameter and trident’s finger diameter of 8 mm. Magnets below 5 mm of diameter will not cope with the magnetic force requirements of the application due to the weight of the complete telescopic system. In this case, a cylindrical neodymium magnet of 6.5 mm diameter and 3 mm height was selected.

**Figure 11:**
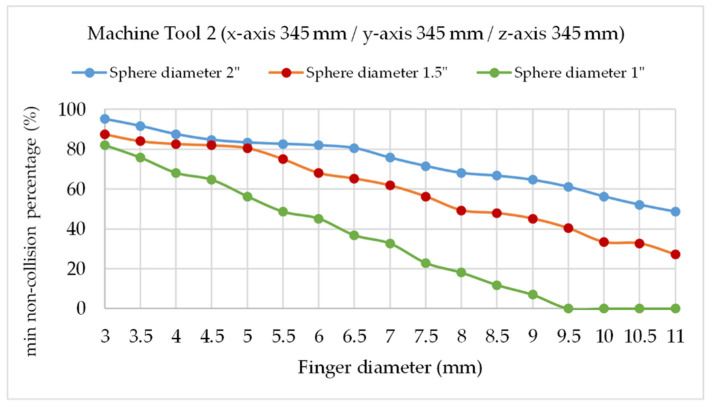
Finger diameter for min 50% non-collision positions in machine tool 2 (sphere diameters 1′′, 1.5′′, and 2”).

The same results are observed for the small-sized machine tool 2 in Figure 11, obtaining maximum finger diameters of 10.5, 8, and 5 mm, respectively, for fixed spheres of 2′′ (50.8 mm), 1.5′′ (38.1 mm), and 1′′ (25.4 mm) diameters. In this case, the same fixed sphere of 1.5′′ diameter and trident’s finger diameter of 8 mm could be suitable for machine tool 2 verification, selecting also a cylindrical neodymium magnet of 6.5 mm diameter and 3 mm height.

## 3. Prototype Assembling of the High Precision Telescopic System

**Figure 12:**
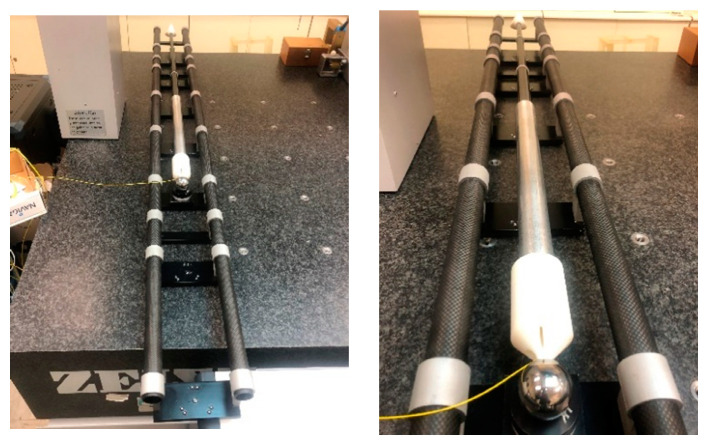
Telescopic arm prototype assembling on calibration artifact.

The design was validated with the assembling of a prototype of a single telescopic arm including the manufacturing of the main components previously presented such as the telescopic system and the trident. An image of the first complete prototype mounted on the length calibration artifact is shown in Figure 12. Future project activities will include the experimental validation and calibration of the system.

## 4. Discussion

The work presents the design of a new high precision telescopic system for small and medium-sized machine tool verification, consisting in three lines with measuring principle based on simultaneous laser multilateration. The system offers high precision because of the interferometric measurement system included and allows the autonomous tracking of a sphere joined to the spindle nose of the MT by simultaneous contact of all the lines. The working principle of the telescopic system is explained and a system design is proposed considering the main design parameters and the application’s requirements. The main contributions of the paper are summarized as following:

- The main advantage of the system is that it allows data capture in a single cycle. This is possible because of the simultaneous operation of the three telescopic arms that are registered simultaneously to a sphere fixed on the MT by means of a newly developed multipoint kinematic coupling (trident).
- There are two concepts developed for the fixation and positioning of the system in the MT. The conventional one includes a fixed sphere on the MT spindle nose and the telescopic system supported on the MT table. The second inverted positioning has the fixed part of the telescopic arms suspended from the MT spindle nose and the fixed sphere located on the MT table. This last set-up allows the verification of the MT rotating axes in a more convenient way.
- In relation with the angle between the telescopic systems in the MTs verification, this study reveals: (i) the fixed points of support of the arms must be arranged in such a way that the angles between the arms are centered at 90 degrees, which is desirable for the precise operation of the multilateration, with maximum values of around 120° and minimum values of 60°; (ii) as the x-travel increases, the minimum angle decreases; (iii) an angle of 15° on the trident body makes it possible to work in a single cycle with verification volumes on machines with unbalanced travels, such as up to 3200 × 600 × 600 mm.
- Differences have been observed between the telescopic system requirements for small and medium-sized machine tools concerning the telescopic arm length. For machine tool 1, its working volume could be verified with a telescopic system with 5 stages (*n*), stage length (*L_i_*) of 500 mm, and 400 mm effective length (*L_ief_*). The maximum (*L_max_*) and minimum (*L_min_*) lengths of the complete telescopic arm would be 2254 and 654 mm, respectively, with an effective measuring range (Δ_L_) of 1600 mm. It is noted that to verify machines with travels greater than 1400 mm, more stages need to be incorporated into the telescopic arm. For MT 2, a 5-stage telescopic system (n) is proposed, with stage length (L_i_) of 223.5 mm and effective length (*L_ief_*) of 146.3 mm. This design presents maximum (*L_max_*) and minimum (*L_min_*) arm lengths of 960 and 375 mm, respectively, with effective measuring range (Δ_L_) of 585 mm, which would be sufficient for the verification volume of the small machine.
- Minimizing the misalignment of the telescopic arm was considered as a requirement in its design, due to its influence on the alignment of the measuring system. To this end, the influence of the radial clearance between stages and the effect of the arm’s own bending on its extension were evaluated.
- It is extremely important for the precise positioning of the arms on the sphere and to avoid collisions between the kinematic couplings, the correct selection of the magnetic system on the trident fingers and its relation to the size of the fixed registration sphere in the MT spindle nose. In the first prototype design, applicable in this case for both MT sizes, we considered a fixed sphere of 1.5′′ diameter and trident’s finger diameter of 8 mm, selecting a cylindrical neodymium magnet of 6.5 mm diameter and 3 mm height because of magnetic force requirements considering the dynamics and the weight of the system.

## Figures and Tables

**Table 1:** Machine tool and measurement equipment specification.

Device	Item	Value	Unit
Machine tool 1	Type	3-axis vertical mill	
	Travel range X axis	1400	mm
	Travel range Y axis	600	mm
	Travel range Z axis	600	mm
Machine tool 2	Type	5 axis vertical mill	
	Travel range X axis	345	mm
	Travel range Y axis	345	mm
	Travel range Z axis	345	mm
	Rotary axis	A/C	
Displacement measuring interferometer	Type	IDS3010	
	DFB laser (infrared)	1530	nm
	Number of axes	3	
	Working distance	5	m
	Max target velocity	2	m/s
	Resolution	1	pm

**Table 2:** Angle between telescopic systems simulation in verification sequence for MT 1 (1400 × 600 × 600 mm).

Verification Points	1	2	3	4	5	6	7	8	9		
*θ*_12_ (°)	90.0	82.7	74.2	122.4	108.1	61.0	59.7	72.9	69.7	122.4	max
*θ*_23_ (°)	90.0	62.0	70.5	75.4	98.0	62.0	70.5	75.4	98.0	59.7	min
*θ*_31_ (°)	90.0	61.0	59.7	72.9	69.7	82.7	74.2	122.4	108.1		
